# Pretreatment with LCK inhibitors chemosensitizes cisplatin‐resistant endometrioid ovarian tumors

**DOI:** 10.1186/s13048-021-00797-x

**Published:** 2021-04-22

**Authors:** Katie K. Crean-Tate, Chad Braley, Goutam Dey, Emily Esakov, Caner Saygin, Alexandria Trestan, Daniel J. Silver, Soumya M. Turaga, Elizabeth V. Connor, Robert DeBernardo, Chad M. Michener, Peter G. Rose, Justin Lathia, Ofer Reizes

**Affiliations:** 1 Department of Gynecologic Oncology, Cleveland Clinic Foundation, Women’s Health Institute, OH Cleveland, USA; 2 Department of Gynecologic Oncology, Sutter Cancer Center, 2800 L Street, Suite 300, CA 95816 Sacramento, USA; 3 Department of Cardiovascular and Metabolic Sciences, Lerner Research Institute, Case Comprehensive Cancer Center, The Laura J. Fogarty Endowed Chair in Uterine Cancer Research, 9500 Euclid Avenue, NC10, OH 44195 Cleveland, USA; 4 Department of Internal Medicine, The Ohio State University, OH Columbus, USA; 5 Department of Gynecologic Oncology, Billings Clinic Cancer Center, MT Billings, USA

**Keywords:** Ovarian cancer, Platinum resistance, LCK inhibitor, Chemosensitization

## Abstract

**Background:**

Ovarian cancer is the most fatal gynecologic malignancy in the United States. While chemotherapy is effective in the vast majority of ovarian cancer patients, recurrence and resistance to standard systemic therapy is nearly inevitable. We discovered that activation of the non-receptor tyrosine kinase Lymphocyte Cell-Specific Protein-Tyrosine Kinase (LCK) promoted cisplatin resistance. Here, we hypothesized that treating high grade, platinum resistant endometrioid cancer cells with an LCK inhibitor (LCKi) followed by co-treatment with cisplatin would lead to increased cisplatin efficacy. Our objective was to assess clinical outcomes associated with increased LCK expression, test our hypothesis of utilizing LCKi as pre-treatment followed by co-treatment with cisplatin in platinum resistant ovarian cancer *in vitro*, and evaluate our findings *in vivo* to assess LCKi applicability as a therapeutic agent.

**Results:**

Kaplan-Meier (KM) plotter data indicated LCK expression is associated with significantly worse median progression-free survival (HR 3.19, *p* = 0.02), and a trend toward decreased overall survival in endometrioid ovarian tumors with elevated LCK expression (HR 2.45, *p* = 0.41). *In vitro*, cisplatin resistant ovarian endometrioid cells treated first with LCKi followed by combination LCKi-cisplatin treatment showed decreased cell viability and increased apoptosis. Immunoblot studies revealed LCKi led to increased expression of phosphorylated H2A histone family X ($\gamma$-H2AX), a marker for DNA damage. *In vivo* results demonstrate treatment with LCKi followed by LCKi-cisplatin led to significantly slowed tumor growth.

**Conclusions:**

We identified a strategy to therapeutically target cisplatin resistant endometrioid ovarian cancer leading to chemosensitization to platinum chemotherapy via treatment with LCKi followed by co-treatment with LCKi-cisplatin.

## Background

Gynecologic malignancy is common, with endometrial and ovarian cancers being the most common incident types in the United States. Ovarian cancer is the most fatal gynecologic malignancy in the United States, with only a 48 % survival at 5 years after diagnosis [1]. Typically, advanced disease in ovarian cancer is treated with cytoreductive surgery and platinum-based chemotherapy. Up to 15 % of ovarian cancers have endometrioid subtype histologically [2]. Unfortunately, high-grade endometrioid cancers prove difficult to treat due to recurrence and chemoresistance [3]. In ovarian cancer, while up to 85 % of patients will enter remission with standard treatment of debulking surgery and platinum-taxane chemotherapy, most of these patients will recur [4]. In the 15 % of patients failing standard therapy, disease persists or progresses within the first six months after chemotherapy, indicating platinum-resistant disease. For those who enter remission, progression to platinum-resistant disease is pervasive [5]. The prognosis is particularly poor in those with platinum-resistant disease, with response rates below 20 % for subsequent lines of chemotherapy and continued decrease in disease free interval with each subsequent therapy [6]. Recurrent ovarian cancer is considered incurable, with goals of care aimed at symptom management with alternative regimens of chemotherapy [7]. Given the poor prognosis in patients with platinum-resistant disease, identification of chemoresistance pathways is necessary for development of therapies to sensitize resistant ovarian cancer [8].

Endometrioid tumors have previously been shown to exhibit a self-renewing population of cells termed cancer stem cells (CSC) [9, 10]. CSCs are associated with both tumor recurrence and chemoresistance in multiple tumor types [9, 11–13]. Previously in the Reizes lab, Saygin et al. identified a novel pathway in CSCs leading to chemoresistance in endometrioid tumors via activation of the non-receptor tyrosine kinase LCK [10]. Saygin et al. utilized pharmacologic inhibition of LCK to incur increased sensitization to cisplatin in endometrioid CSCs. This finding was verified with LCK-silenced CSCs via shRNA (short hairpin RNA) constructs. Additionally, LCK overexpression in non-CSCs indicated higher survival rates and lower apoptosis rates compared to an empty vector control [10]. *In vitro* studies using saracatinib, an investigational drug that inhibits LCK, showed sensitization of cisplatin resistant endometrioid cells to cisplatin in a CSC population. With inhibition of LCK, DNA repair genes were attenuated and cells showed increased sensitivity to cisplatin [10]. Phase I studies of saracatinib utilized in multiple cancer types have indicated an appropriate safety profile, though follow up randomized human trials in ovarian cancer have fallen short in translating the effect into clinical use [14, 15]. With our initial results and proposed mechanism of action of chemosensitization, we hypothesized that treating cancer cells first with an LCK inhibitor, followed by co-treatment with an LCK inhibitor and cisplatin, would enhance the chemosensitization effect. Our primary objective was to test this hypothesis *in vitro* in a cisplatin resistant endometrioid cancer model, followed by *in vivo* as a proof of concept.

## Results

### LCK expression is associated with poor patient survival

**Fig. 1:**
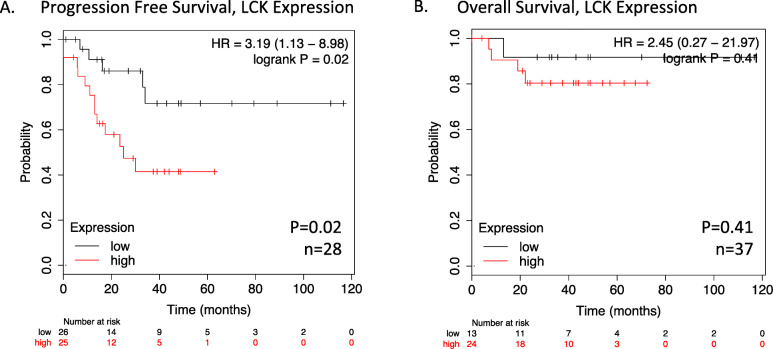
LCK expression is associated with poorer patient survival. Kaplan-Meier progression-free and overall survival curves were obtained from Kaplan-Meier Plotter (KM Plotter: http://kmplot.com/analysis/) for endometrioid ovarian cancer patients who had high versus low tumor mRNA expression of LCK (**a, b**) prior to therapy

Given the previously described mechanism of cisplatin resistance via the LCK pathway, we hypothesized that increased LCK expression would be associated with worse clinical outcomes. We assessed survival outcomes with increased LCK expression for endometrioid ovarian cancer using Kaplan-Meier Plotter database (KM Plotter: http://kmplot.com/analysis/). The database was queried for patients of all stages and grades with endometrioid ovarian cancer. In endometrioid ovarian cancer, LCK expression is associated with significantly worse median progression-free survival (HR 3.19, *p* = 0.02, Fig. 1a). Overall survival is not significantly different between groups, though a non-significant trend toward decreased survival was seen with HR 2.45 (*p* = 0.41, Fig. 1b). This is also supported by gene expression profiling data from Tothill et al. that found an over 2-fold increased expression in LCK in ovarian cancer subtypes containing high grade endometrioid cells and associated significantly decreased PFS (*p* < 0.001) and OS (*p* < 0.001) in this subtype [16]. This compares with half the gene expression of LCK in the predominantly low grade endometrioid ovarian cancer subtype, which showed the most favorable PFS and OS [16]. These data indicate increased LCK expression in endometrioid ovarian cancer correlates with poorer clinical outcomes.

### Pretreatment with LCK inhibitors chemosensitize cisplatin resistant endometrioid cells and increase apoptosis

**Fig. 2:**
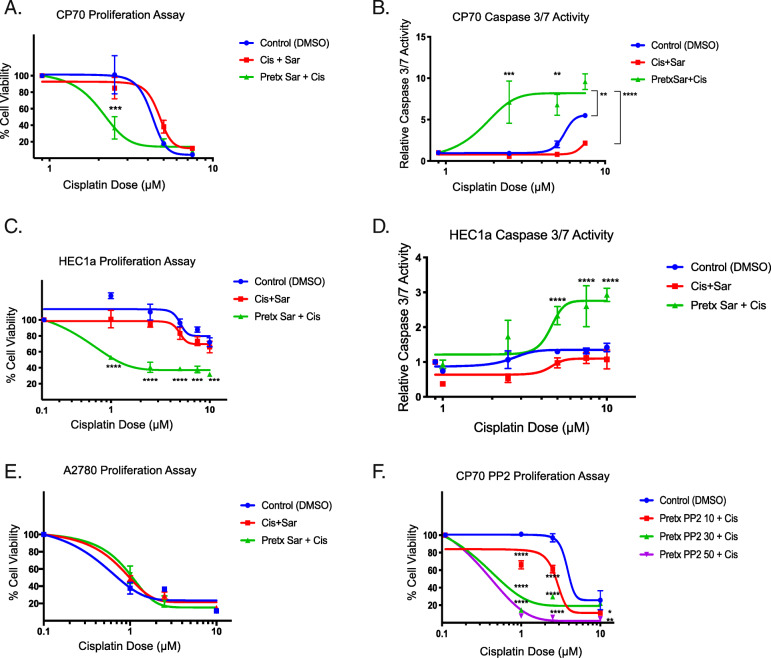
LCK inhibitors chemosensitize cisplatin resistant endometrioid cells and increase apoptosis. Cisplatin resistant ovarian endometrioid cells (CP70) were cultured and pretreated with an LCK inhibitor (saracatinib) or vehicle, followed by vehicle or combination LCKi-cisplatin, followed by cell viability assay performed with the CellTiterGlo Assay (**a**). Caspase 3/7 Assay was then performed to assess apoptosis (**b**)**.** A second cisplatin resistant endometrioid cell line (HEC1a) was similarly treated and tested with subsequent proliferation and apoptosis assays performed (**c, d**)**.** Cisplatin sensitive ovarian endometrioid cells (A2780) were cultured and treated according to the aforementioned paradigm (**e**). An alternative LCK inhibitor (PP2) was utilized for pretreatment in CP70 cells followed by co-treatment with PP2-cisplatin (**f**)**.** All data represent at minimum three independent experiments with three technical replicates

In our prior studies, LCK inhibition in the ovarian endometrioid CSC population led to increased cisplatin sensitivity [10]. Given that CSCs are known to be a chemoresistant population closely associated with disease recurrence, we hypothesized that inhibition of the LCK pathway would lead to sensitization of platinum resistant endometrioid cells. We performed an *in vitro* cellular proliferation assay in cisplatin resistant ovarian endometrioid cells (CP70) treated with vehicle or the LCK inhibitor (LCKi) saracatinib followed by assessing chemosensitivity to cisplatin via dose response. There was no significant difference with co-treatment of LCKi and cisplatin alone, so we hypothesized that pretreatment with LCKi is required to effectively sensitize these cells to cisplatin. CP70 cells pretreated with saracatinib and then treated with combination saracatinib-cisplatin exhibited significantly reduced proliferation (Fig. 2a). In parallel, we tested the effect of saracatinib-cisplatin treatment on apoptosis using Caspase Glo. We determined that CP70 cells pretreated with saracatinib followed by cisplatin plus saracatinib led to increased apoptosis compared to no pretreatment (Fig. 2b). These findings were replicated in HEC1a, a cisplatin resistant endometrioid endometrial cancer model. HEC1a pretreated with saracatinib followed by cisplatin co-treatment with saracatinib exhibited inhibition of cell viability (Fig. 2c) and increased apoptosis (Fig. 2d) compared to vehicle treated cells.

To validate our hypothesis that treatment with LCKi followed by co-treatment with cisplatin leads to decreased proliferation specifically in cisplatin resistant endometrioid cells, we performed a proliferation assay in A2780 cells, the chemo-naive parent cell line of CP70. We found that pretreatment with saracatinib, as well as simultaneous treatment with saracatinib and cisplatin, did not alter cell viability (Fig. 2e). To validate our hypothesis that saracatinib chemosensitizes these cells via LCK inhibition, we pretreated CP70 cells with a more selective LCKi, PP2, followed by cisplatin co-treatment, and found a similar left shift in dose response compared to control groups treated with cisplatin alone (Fig. 2f). These data indicate that platinum resistant endometrioid cells pretreated with LCKi followed by co-treatment with LCKi and cisplatin show decreased cell proliferation and increased apoptosis.

### Cisplatin resistant endometrioid cells treated with LCK inhibitors lead to increased DNA double strand breaks

**Fig. 3:**
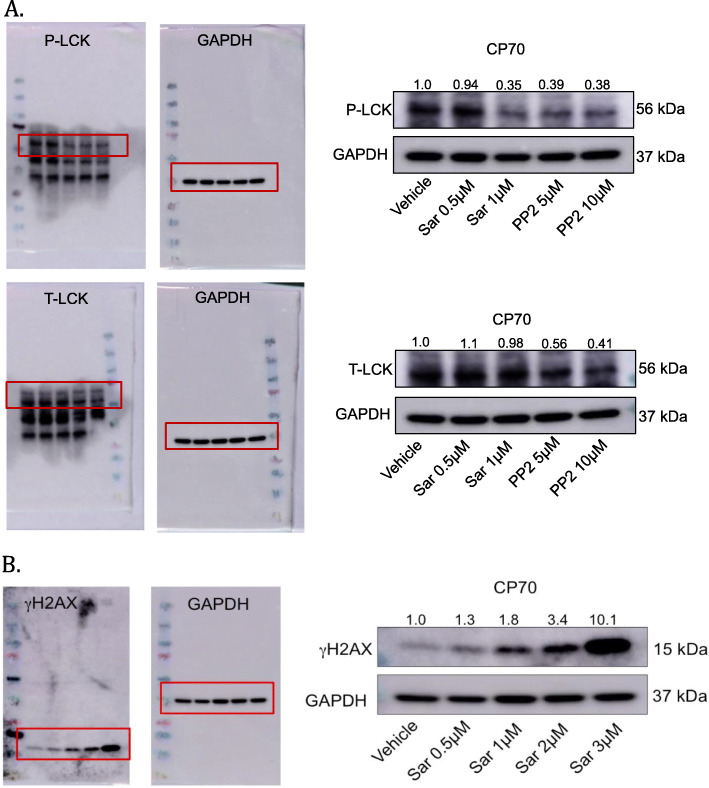
Cisplatin resistant endometrioid cells treated with LCK inhibitors indicate decreased P-LCK and ovarian endometrioid cells treated with LCK inhibitor indicate increased DNA double strand breaks. Cisplatin resistant ovarian endometrioid cancer cells (CP70) were treated with DMSO, LCK inhibitor saracatinib (Sar) or PP2 at indicated doses for 48 h. Protein lysates were then immunoblotted for phosphorylated LCK (P-LCK Y394) and total LCK (T-LCK). Fold changes of protein expression are shown in the figure, with values normalized to vehicle control. GAPDH was used as loading control (**a**). CP70 cells treated with the indicated varied doses of saracatinib (Sar) were immunoblotted for 훾H2AX. Fold changes of protein expression are shown in the figure, with values normalized to vehicle control. GAPDH was used as a loading control (**b**). Each experiment was performed with at least three technical replicates

To investigate the mechanism by which LCK inhibitors decrease cisplatin resistance, we tested whether LCK inhibitors decrease phosphorylation of LCK in CP70 cells by immunoblot. We found that Y394 phosphorylated LCK (P-LCK) was significantly reduced when cells are exposed to LCK inhibitors saracatinib or PP2. Y394 P-LCK is the autophosphorylation site on LCK necessary for kinase activation and function [17]. Total LCK (T-LCK) was unchanged in vehicle and saracatinib-treated cells, whereas there was a reduction in PP2-treated cells. GAPDH was used as a loading control (Fig. 3a). We tested for DNA damage by blotting for 훾H2AX, a marker of DNA double strand breaks based on phosphorylation of histones [18, 19]. We found an increase in 훾H2AX in LCKi treated CP70 cells (Fig. 3b).These data indicate that endometrioid ovarian cancer cells exposed to LCKi demonstrate a decrease in phosphorylated LCK and an increase in DNA double strand breaks.

### Treatment with LCK inhibitor followed by co‐treatment with cisplatin decreases tumor growth in vivo

**Fig. 4:**
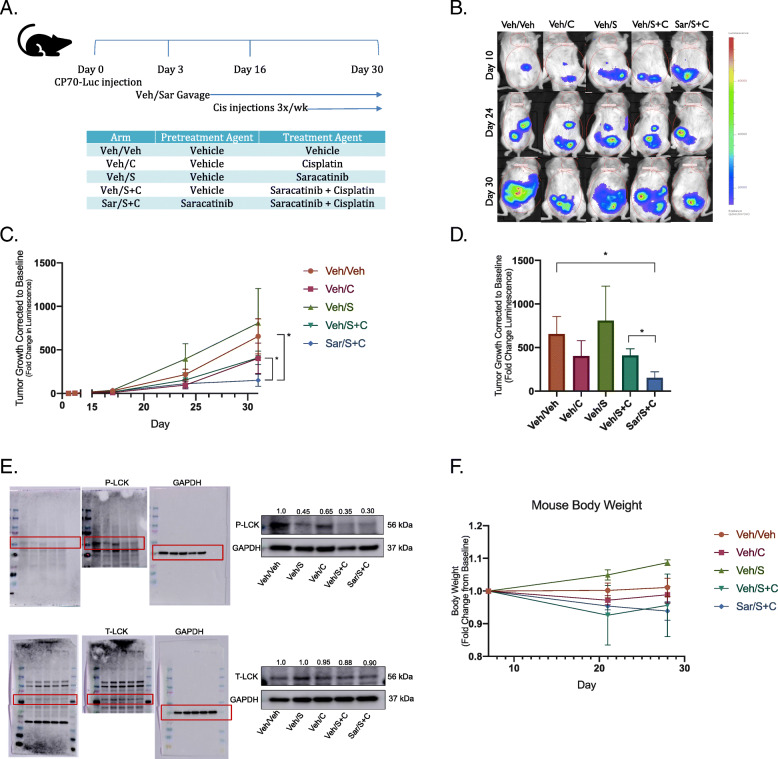
Pretreatment with LCK inhibitor followed by LCKi-cisplatin treatment attenuates tumor burden, and treated tumors indicate decreased P-LCK. NSG mice were injected with CP70-luciferase transfected cells followed by pretreatment with LCKi (6 mice) or vehicle (24 mice) for 14 days. LCKi mice were then co-treated with LCKi and cisplatin, and vehicle mice were randomized to further treatment with vehicle, cisplatin, saracatinib, or combination (6 mice per arm) (**a**). IVIS imaging was obtained on a weekly basis to assess tumor growth (**b**). IVIS luminescence was corrected to baseline for each arm and assessed over time (**c**) and at the experimental endpoint (**d**). Tumors from NSG mice were extracted, and protein lysates were prepared and immunoblotted for protein expression of P-LCK (Y394) and T-LCK. Fold changes of protein expression are shown in the figure, with values normalized to vehicle control. GAPDH was used as a loading control (**e**). Two tumors per condition were probed, with at least three technical replicates performed. Mouse body weight at day 7, 21, and 28 were obtained for each arm, with weights shown as fold change from baseline (**f**)

Given the *in vitro* findings, we next tested our hypothesis in an *in vivo* model. We injected NOD severe combined immunodeficient (SCID) IL2R gamma (NSG) mice with CP70 cells virally transduced with luciferase and utilized the *in vivo* imaging system (IVIS) to detect tumor growth on a weekly frequency. After injection of tumor cells (day 0), mice were placed in one of two arms: pre-treatment with saracatinib or vehicle via oral gavage three days per week, initiated on day 3. On day 16 when all mice were confirmed to have detectable tumor on IVIS, mice pretreated with saracatinib were injected with cisplatin three times weekly, and mice pretreated with vehicle were randomly assigned to one of four arms: cisplatin, saracatinib, combination cisplatin and saracatinib, or vehicle alone, given three times per week. Mice were then euthanized on day 30 (Fig. 4a). Images presented for each subgroup were taken from the same mouse over time. Mice treated first with vehicle followed by cisplatin, saracatinib, or combination cisplatin and saracatinib all showed a steady increase in tumor burden over time. However, in mice first treated with saracatinib followed by combination cisplatin and saracatinib therapy, tumor growth appeared stable or attenuated (Fig. 4b, c). At the experimental endpoint (Day 30), tumor growth in the vehicle group was similar to cisplatin, saracatinib, and combination cisplatin and saracatinib arms. In contrast, pretreatment with saracatinib followed by combination saracatinib-cisplatin treatment exhibited a significant reduction in tumor growth compared to vehicle as well as combination alone (Fig. 4d). These findings were also reflected in the mouse body weight, with greatest weight increase observed in vehicle and saracatinib arms, and the largest reduction in weight, though nonsignificant (*p* = 0.059), observed in the pretreatment saracatinib arm (Fig. 4f). Of note, this data represents tumor growth corrected to baseline, not absolute tumor size, indicating that the rate at which tumor growth is occurring is significantly reduced relative to other treatment groups. These data demonstrate that pretreatment with LCKi followed by LCKi-cisplatin co-treatment leads to decreased tumor burden in cisplatin resistant endometrioid ovarian cancer *in vivo*.

### Treatment with LCK inhibitor in vivo leads to decreased P-LCK in cisplatin‐resistant endometrioid tumors

To validate the efficacy of LCKi, we assessed Y394 P-LCK expression in tumors extracted from our *in vivo* study. We found that P-LCK in tumors from mice treated with saracatinib or a combination of saracatinib and cisplatin expressed lower P-LCK than mice treated only with vehicle or cisplatin. T-LCK did not exhibit a significant reduction in expression. GAPDH was used as a loading control (Fig. 4e). Thus, LCKi was efficacious at inhibiting LCK activation.

## Discussion

Despite most ovarian cancers displaying an excellent initial response to standard chemotherapy, the majority of advanced stage patients recur, with eventual resistance to our most effective chemotherapy agents. This pattern of pervasive relapse and ensuing chemoresistance is the cause for the poor survival rates seen in ovarian cancer today [3, 5, 9]. Studies have focused on identifying a targetable pathway promoting chemoresistance in order to reduce recurrence [9, 11, 12]. Previous studies by Saygin et al [10] identified a novel pathway leading to chemoresistance in endometrioid tumors in which CD55 mediated DNA repair via phosphorylation of LCK. We assessed clinical outcomes associated with LCK expression. We found that high LCK expression predicted a significant effect on PFS with a three-fold increase in survival from 13 months to 34 months in low versus high LCK expressing tumors (Fig. 1). This data suggests a clinical benefit to addressing tumors with increased LCK expression, and thus a potential targetable pathway in recurrent ovarian endometrioid tumors.

Standard chemotherapy in ovarian cancer includes a platinum and taxane agent, and survival decreases as response to platinum therapy diminishes. Prior studies have found that cisplatin resistance is seen with multiple pathways, including increased DNA repair enzyme expression and associated reduction in DNA adducts [9, 10, 20]. Through LCK inhibition, DNA repair enzyme expression is attenuated, one of the known pathways to cisplatin resistance [10]. Given this anticipated initial chemosensitization step, we pursued pretreatment with LCK inhibitors followed by co-treatment with cisplatin and found that this technique was effective in decreasing cancer cell populations and increasing apoptosis *in vitro* (Fig. 2). We verified the effects of inhibition of LCK on DNA damage and found an increase in DNA adduct formation with LCK inhibition in immunoblot studies (Fig. 3), an indication that targeting this pathway allows platinum therapy to function in a previously cisplatin resistant cell population.

**Fig. 5:**
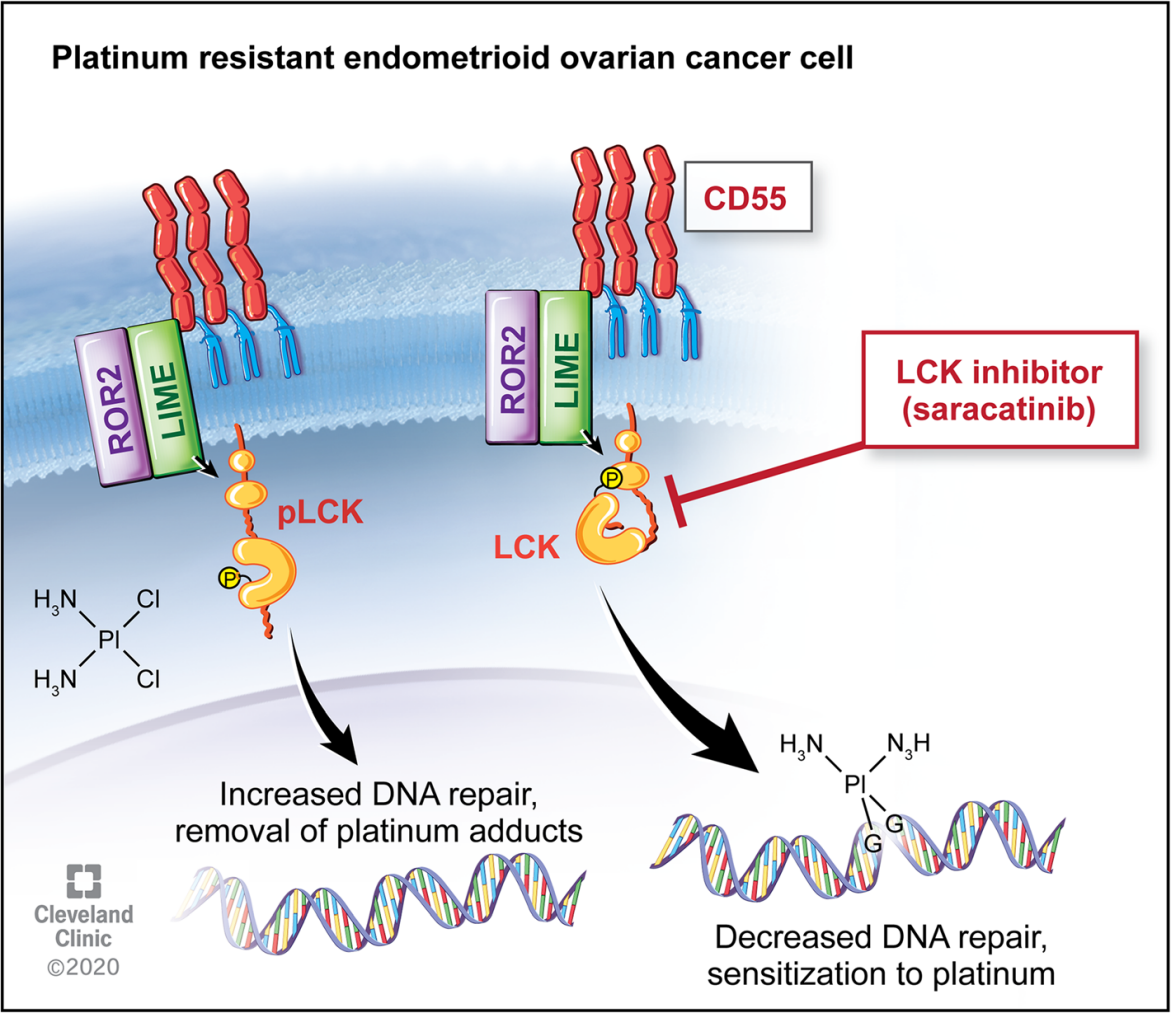
LCK pathway regulates cisplatin resistance in endometrioid tumors. Downstream of CD55, LCK stimulates expression of DNA repair genes, leading to cisplatin resistance. This targetable pathway identifies LCK inhibitors as adjunctive therapy for platinum resistant ovarian endometrioid cancer

A common challenge in translational research is that while *in vitro* studies may prove promising, translating this to effective *in vivo* studies and clinical trials can prove difficult. Saracatinib, an investigational LCKi, has been studied for several cancer types, with mixed results. Studies on safety found appropriate dosing for saracatinib in humans for effective pharmacodynamics while limiting toxicity, indicating this drug would be tolerable in clinical trials [14]. While utilizing saracatinib as monotherapy has not proven efficacious, combination therapy has yielded more promising results. In a study combining saracatinib with carboplatin and/or paclitaxel in solid tumors, objective responses were seen in ovarian, breast and skin cancers, with longest response durations seen in patients with ovarian cancer [14]. However, a randomized trial further assessed treatment with saracatinib in combination with weekly paclitaxel in platinum-resistant ovarian cancer, and found that co-treatment of saracatinib with weekly paclitaxel did not improve outcomes [15]. Of note, the majority of these tumors were serous histology, and patients received only weekly Taxol without platinum in addition to saracatinib. There is no clinical randomized data assessing saracatinib with cisplatin use in platinum resistant patients. We see from this clinical data that saracatinib is well tolerated and may have a role in combination therapy in platinum resistant disease. We tested this hypothesis with a novel administration of saracatinib followed by co-treatment with cisplatin and found a decreased rate of tumor growth *in vivo* (Fig. 4), identifying a targetable pathway (Fig. 5) and providing a novel therapeutic regimen for platinum resistant ovarian endometrioid carcinoma.

This study’s strength lies in the proof of concept findings using both *in vitro* and *in vivo* models. Additionally, whereas prior studies focused on a specific cell population, cancer stem cells, this study utilized a more heterogeneous cell population, more closely simulating a typical tumor microenvironment. Further investigation should also be performed in additional histologic types such as serous and clear cell, as well as substitution of cisplatin for co-treatment with carboplatin, a commonly used platinum agent. Patients with platinum resistant disease have often received multiple lines of chemotherapy previously and would benefit from further treatment options. Given our promising findings, further studies are indicated to pursue LCK inhibitors as an adjunctive therapy to platinum resistant disease in clinical trials.

## Conclusions

In summary, we identified a targetable pathway for chemosensitization of platinum resistant ovarian endometrioid cancer. We found that pretreatment with LCK inhibitors followed by co-treatment with cisplatin leads to decreased cell viability and increased apoptosis *in vitro*. This is associated with increased DNA adduct formation and significantly reduced tumor growth *in vivo*. Further studies are needed to assess the mechanisms behind the enhanced efficacy of pretreatment, as well as further investigation of LCK inhibitors as adjunctive therapy for platinum resistant endometrioid ovarian carcinoma, including other histological subtypes.

## Methods

### Cell culture

Ovarian endometrioid adenocarcinoma cell lines A2780 (cisplatin sensitive) and its cisplatin resistant daughter cell line CP70 were cultured in Dulbecco’s Modified Eagle Medium (DMEM) supplemented with 10 % heat-inactivated fetal bovine serum at 37 °C in a humidified atmosphere in 5 % CO_2_. Cisplatin resistant endometrioid endometrial cancer cell line HEC1a was cultured in modified McCoy’s 5a medium supplemented with 10 % heat-inactivated fetal bovine serum, also at similar conditions. Cell lines were obtained from the Cleveland Clinic centralized research core facility, through which cell lines were previously obtained from the American Type Culture Collection (ATCC) and authenticated. At approximately 80 % confluence, trypsin (0.25 %)/EDTA solution or Accutase was used to lift cells for passaging as needed for continued experiments until passage 10, at which point a fresh allotment of cells was plated. Cisplatin was obtained from Cleveland Clinic Hospital pharmacy, with 1 mg/mL stock solutions stored at room temperature protected from light given its photosensitivity. Saracatinib (AZD0530) was purchased from Selleck Chemicals, dissolved in DMSO (Sigma Cat#D2650), and 10 $\mu$M stock solutions were aliquoted and stored at -20 °C. PP2 (AG1879) was purchased from Selleck Chemicals, dissolved in DMSO (Sigma Cat#D2650), and 10 $\mu$M stock solutions aliquoted and stored at -20 °C. Mycoplasma testing was performed and negative.

### Proliferation assays and caspase 3/7 assays

The appropriate cancer cells for each experiment were pre-treated with saracatinib (1$\mu$M), PP2 (10–50 $\mu$M), or vehicle (DMSO at similar concentration to drug of interest) for 4 days in T75 flasks. Cells were then plated in 96-well plates at 5,000 cells/well on seeding Day 0, manually counted by hemocytometer using Trypan blue dye exclusion as live cell marker. Cisplatin was then applied the next day at doses of 0–10 $\mu$M, with/without saracatinib, PP2 or vehicle, and treatment was ongoing for 4 to 6 days. Measured proliferation was assessed by CellTiter-Glo (Promega, Southampton, UK) as per manufacturer’s instructions. Percentage survival was normalized to the untreated control for each group.

Caspase 3/7 Assay kit (Promega, Southampton, UK) was utilized to assess apoptosis as per manufacturer’s instructions. This was performed alongside CellTiter-Glo to correct for viable cell density. Relative Caspase activities were normalized to untreated controls in each group, with activity assessed from 30 to 120 min. Three independent experiments were performed at minimum, each with three technical replicates.

### Immunoblotting

Protein lysates were obtained with cell lysis in 20mM Tris-HCl (pH 7.5), 150mM NaCl, 1 mM Na2EDTA, 1 % NP-40, 1 mM EGTA, 1 % sodium pyrophosphate, 1 mM β-glycerophosphate, 1mM sodium orthovanadate, 1 $\mu$g/mL leupeptin, 20 mM NaF and 1 mM PMSF. Protein concentrations were measured with BCA Protein Assay Kit (ThermoFisher Scientific). Protein concentrations of 40$\mu$g of total protein were resolved in 10–12 % SDS-PAGE and transferred to PVDF membrane. Membranes were incubated overnight at 4 °C with primary antibodies against P-LCK (Y394) (1:1000) (R&D Systems, Catalog#MAB7500, Clone#755,103), T-LCK (1:1000) (Proteintech, Catalog#12477-1-AP), GAPDH (1:1000) (Proteintech, Catalog#HRP-60,004), and $\gamma$-H2AX (1:1000) (Cell Signaling, Catalog#2257). Secondary anti-mouse or anti-rabbit IgG antibodies conjugated to horseradish peroxidase (HRP) (1:3000) (Cell Signaling, Catalog#7076) or (1:25,000) (ProMega, Catalog#W4011) were used. ECl (Pierce) was then used to visualize immunoreactive bands.

### In vivo study

All animal procedures were evaluated and approved prior to initiation by the Institutional Animal Care and Use Committee (IACUC) of the Cleveland Clinic Lerner Research Institute. NOD severe combined immunodeficient (SCID) IL2R gamma (NSG) mice were purchased from the Biological Response Unit (BRU) at the Cleveland Clinic and housed in microisolator units under IACUC protocol #2018 − 1940. Thirty mice were injected intraperitoneally with 1 million CP70-luciferase virally transduced cells. At the time of injection (day 0), mice were placed in one of two arms, which started day 3: six mice began receiving pre-treatment with saracatinib (Selleck), 25 mg/kg dissolved in 0.5 % hydroxypropyl methylcellulose (Sigma-Aldrich), 0.1 % Tween 80 (Sigma-Aldrich) via oral gavage three days per week, and 24 mice received vehicle via oral gavage on the same schedule.

Bioluminescence images to detect tumor burden were taken with Xenogen *in vivo* imaging system (IVIS, PerkinElmer) using D-luciferin as previously described [21]. Mice received an IP injection of D-luciferin (Goldbio LUCK-1G, 150 mg/kg in 150$\mu$L) under inhaled isoflurane anesthesia. Images were analyzed (Living Image Software) and bioluminescence plots of photon flux (photons/second/cm^2^/steradian) over time were computed for each mouse, with normalization against day 0 signal values. Non-tumor and black backgrounds were also subtracted from each tumor burden region of interest. All images were obtained with a 15 s exposure. On day 16 when all mice were confirmed to have tumor by IVIS, mice pretreated with saracatinib were also treated with cisplatin (2.5 mg/kg, 3 times per week) injected intraperitoneally, as previously described [10]. On day 16, mice pretreated with vehicle were randomly assigned to one of four arms (6 mice per arm), whereby they were treated with cisplatin, saracatinib, combination cisplatin and saracatinib, or vehicle alone. Mice were sacrificed on day 30 and all visible tumor was collected for future studies. There were no significant observable treatment toxicities. All mouse procedures were performed under adherence to protocols approved by the Institute Animal Care and Use Committee at the Lerner Research Institute, Cleveland Clinic.

### Statistical analysis

Statistical analysis was calculated by one-way ANOVA and two sample t-test, with *p*-values included. Statistical significance is denoted via * to represent p-value of < 0.05 but > 0.01, ** representing *p*-value of < 0.01 but > 0.001, and ** representing *p*-value < 0.001. For proliferation assays, IC50 was calculated using nonparametric values set to nonlinear fit curve as per statistical analysis performed with GraphPad Prism. Survival data was obtained from Kaplan-Meier Plotter (KM Plotter: http://kmplot.com/analysis/) for endometrioid ovarian cancer based on CD55 and LCK mRNA expression. KM Plotter survival data is obtained from an online database collected from The Cancer Genome Atlas (TCGA), Gene Expression Omnibus (GEO) and European Genome-phenome Archive (EGA).

## Data Availability

The KM plotter dataset is available at https://kmplot.com/analysis/index.php?p=service&cancer=ovar. The datasets used and analyzed for the current study are available from the corresponding author upon reasonable request.

## Declarations

### Author’s Information

OR and PGR are co-directors of the Center for Research Excellence in Gynecologic Cancer at the Cleveland Clinic.
